# Palatable disruption: the politics of plant milk

**DOI:** 10.1007/s10460-020-10022-y

**Published:** 2020-01-30

**Authors:** Nathan Clay, Alexandra E. Sexton, Tara Garnett, Jamie Lorimer

**Affiliations:** 1 School of Geography and the Environment, University of Oxford, South Parks Road, Oxford, OX1 3QY UK; 2 Food Climate Research Network, Oxford, UK; 3 Environmental Change Institute, University of Oxford, South Parks Road, Oxford, OX1 3QY UK

**Keywords:** Alternative food network, Dairy, Food industry, Neoliberal, Protein, Vegan

## Abstract

Plant-based milk alternatives–or *mylks*–have surged in popularity over the past ten years. We consider the politics and consumer subjectivities fostered by mylks as part of the broader trend towards ‘plant-based’ food. We demonstrate how mylk companies inherit and strategically deploy positive framings of milk as wholesome and convenient, as well as negative framings of dairy as environmentally damaging and cruel, to position plant-based as the ‘better’ alternative. By navigating this affective landscape, brands attempt to (re)make mylk as simultaneously palatable and disruptive to the status quo. We examine the politics of mylks through the concept of *palatable disruption*, where people are encouraged to care about the environment, health, and animal welfare enough to adopt mylks but to ultimately remain consumers of a commodity food. By encouraging consumers to reach for “plant-based” as a way to cope with environmental catastrophe and a life out of balance, mylks promote a neoliberal ethic: they individualize systemic problems and further entrench market mechanisms as solutions, thereby reinforcing the political economy of industrial agriculture. In conclusion, we reflect on the limits of the current plant-based trend for transitioning to more just and sustainable food production and consumption.

##  The rise of plant milks

“If you want to change the world change your milk” (Plenish Drinks 2019).

“The subtle sweet and creamy flavour of Alpro Soya will brighten any breakfast. It isn’t plain, it’s plain delicious!” (Alpro 2019).

Plant-based milk alternatives (or *mylks*^1^) are booming. In the US, sales rose by 61% between 2012 and 2017 (Mintel 2018), reaching $1.9 billion by 2019 (Good Food Institute 2019). Varieties have expanded beyond the traditional soymilk to include mylks made from almond, oat, coconut, pea, hemp, and other grains, seeds, nuts, and legumes. Mylks now account for 13% of total retail milk sales in the US (Good Food Institute 2018) and around 8% in the UK (Mintel 2019). Other plant-based dairy substitutes (ice cream, yogurt, creamer, and cheese) have seen similarly rapid growth, with US sales doubling over the past 2 years to $920 million in 2019 (Good Food Institute 2019).

Once sidelined in natural food stores and health food aisles, plant mylk has ‘gone mainstream,’ as a recent piece in *The Economist* affirms, proclaiming 2019 ‘the year of the vegan’ (Parker 2018). Yet, the recent surge of plant-based milk and meat may owe less to people adopting vegan diets and more to the emerging *flexitarian* trend (Wohl 2019). Flexitarians are people actively reducing meat and dairy consumption for environmental, ethical, and health concerns (Wood 2019). Fittingly, these are the cares promoted by mylk marketing.

This article considers the politics surrounding this mainstreaming of plant-based products to question how mylks are positioned as alternative to dairy milk. By exploring the narrative framings employed to position mylks as the better milk, we consider the consumer subjectivities fostered and the political economy that this reinforces. We examine the politics of plant milk by developing the concept of *palatable disruption*, which posits that people are encouraged to care about the environment, health, and animal welfare enough to adopt mylks but to ultimately remain consumers of a commodity food. The rise of flexitarianism marks a change in how many people see their relationships to the environment through food. While this could have important implications for sustainability, we argue that it has created an opportunity for the food industry to reposition milk as a fix to environmental and health crises caused by overproduction. We follow work on alternative food networks (AFNs) such as local, organic, and fair trade (DuPuis and Goodman 2005; Goodman 2004; Guthman 2008) to critically assess the politics enabled by the rise of mylks. Our motivation is to explore how plant-based foods have been de-politicized and naturalized as solutions to climate change, animal welfare, and human health challenges. Our analysis is not meant to be dismissive but to urge caution against any implicit assumption that plant-based offers food futures that are better for the environment, health, and animal welfare.

### Dairy crisis

The rise of mylks comes at a particularly fraught moment for the dairy industry. Dairy is experiencing a pronounced economic crisis as a result of overproduction and decreasing consumer demand (Clay et al. 2020). After 50 years of policies pushing dairy intensification and retailer-controlled milk pricing,^2^ profit margins for milk are extraordinarily thin. Production costs (including feed, land, and water) have ramped up in recent years (Hadrich et al. 2017) and dairy farm concentration has accelerated over the past decade, with thousands of smaller farms in the US and Europe going out of business every year and herd sizes on larger farms growing exponentially (Clay et al. 2020). Fluid milk consumption has been declining since the 1970s in the US and UK. Fluid milk consumption in the UK is about half of 1970s levels (Defra 2017). Consumption is particularly low among younger generations. In the UK, only 73% of people aged 16 to 24 now drink milk, compared to 92% of those over 45 years (Mintel 2019). From 2017 to 2018, fluid dairy milk sales in the US declined by 8%, a loss of $1.1 billion (Dairy Farmers of America 2019). Mylk sales increased by 9% that year (Plant-Based Foods Association 2018).

One driver of decreased dairy consumption is that people—particularly younger generations (ages 16 to 24)—increasingly associate dairy farming with environmental degradation (Mintel 2019). Recent studies reveal a large water, land, and greenhouse gas footprint of dairy relative to other foods (Poore and Nemececk 2018; Springmann et al. 2018). Others suggest that reducing consumption of animal protein may both decrease human mortality and reduce environmental impacts (Westhoek et al. 2014; Springmann et al. 2016; Clark et al., 2019). This story of dairy’s environmental impacts has circulated widely in the UK and US. It was covered by eight articles in *The Guardian* in 2018, including an article headlined “avoiding meat and dairy is the single biggest way to reduce your impact on earth” (Carrington 2018), which ran on the front page and at one time amassed more than 900,000 shares via social media. In short, scientific research and public messaging about the multiple benefits of reducing meat and dairy consumption has never been stronger.

**Fig. 1:**
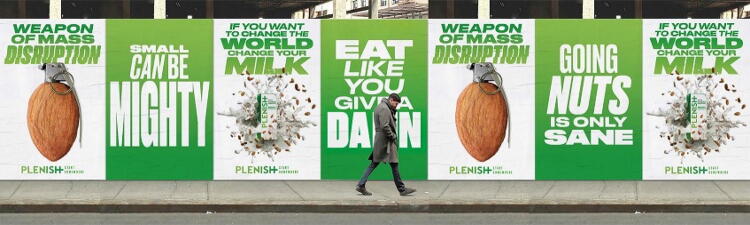
PlenishDrinks advertisement

The ascent of plant mylks has been propelled and shaped by sizeable marketing investment, much of which speaks to these environmental and health concerns. The excerpts opening this paper are taken from cartons of oat and soymilk. These quotes capture the spectrum of current narratives that are used by companies to position mylks as the better alternative to milk. One significant story presents plant mylks as a *disruption*. The UK company Plenish Drinks tells us that ‘if you want to change the world change your milk’ (Fig. 1). This slogan appeared alongside images of milky explosions and an almond taking the form of a hand grenade: a ‘weapon of mass disruption’. Similarly, the Swedish company Oatly ran a marketing campaign that foretells of the rise of a ‘Post Milk Generation’: ‘a non-profit mindset that works to inform the public about the health and sustainability advantages of eating a plant-based diet’.

**Fig. 2:**
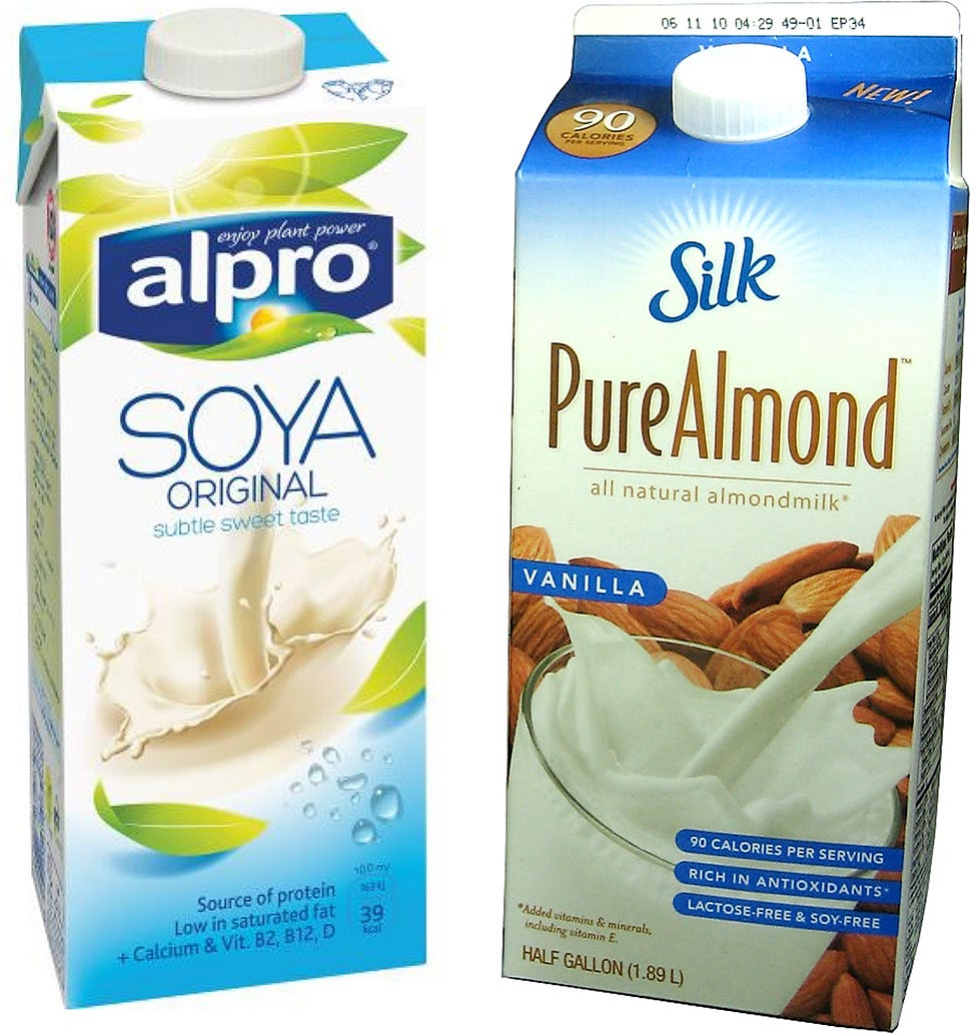
Alpro and silk packaging

In contrast, the formulations of alternativeness by longstanding mylk brands such as Alpro and Silk (both owned since 2017 by the dairy multinational Danone) are comparatively docile. The second quote, on a carton of Alpro soymilk, captures this more measured approach. The language mobilizes inherited framings of milk as wholesome, promising a creamy texture, sweet taste, and familiar role in a convenient breakfast. Alpro’s and Silk’s imagery of flowing white liquid (Fig. 2) and their wide availability in supermarket dairy aisles celebrates mylk’s continued milkiness.

### The politics of plant milk

AFNs such as organic, local, and community supported agriculture were established to counteract problems linked to globalized industrial agriculture. Such alternatives often seek to reformulate social and ecological relations underlying food production, distribution, and consumption to rebuild trust that had been eroded under corporate food regimes (Goodman et al. 2012). In contrast, mylks have emerged as an alternative that is already conspicuously within the food industry. The rapid scaling up and corporate control of plant-based testifies to this. Mylk companies are attracting investments from the likes of Goldman Sachs and from venture capital firms on the order of tens of millions of USD (Fields 2019). The plant-based trend is celebrated by one prominent investor network as indication of ‘an appetite for disruption’ (Ramachandran et al. 2019). Yet, even though mylk brands have proliferated, the majority of market share is concentrated with a few large beverage-focused multinationals—most prominently dairy giant Danone through its Whitewave/Alpro holdings. Danone recorded over $1.9 billion in plant-based beverage sales in 2018 and has promised to triple sales within 5 years (Camacho 2018), a goal that attracted a flurry of investor interest in 2019. The Coca-Cola Company is expanding mylk offerings through their Innocent and AdeS brands. PepsiCo launched an ‘Oat Beverage’ in 2019 through their Quaker brand.

In this paper, we examine how the plant mylk sector employs narrative frames and affective sensibilities to shape food palatability, re-make milk as plant-based, and responsibilize ‘ethical’ consumers. We critically explore how mylks are positioned as the ‘better milk’ through simultaneously securing past framings of milk as ‘good’ (wholesome, healthy, tasty, and convenient) while mobilizing narratives of dairy as ‘bad’ (environmentally damaging and cruel). In different ways, mylks navigate these contrasting narratives with their marketing campaigns. As with AFNs such as organic, fair-trade, or local, mylk’s claims of alterity are founded on a range of cares, including for the environment, bodily health and ‘wellness,’ animal welfare, taste, and convenience. As we will argue in this paper, mylk companies deploy these cares in ways that uphold the political economy of industrial agriculture and grant food industry further power to shape food futures.

We explore the politics of mylks through the concept of palatable disruption. This builds on work by Jesse Goldstein (2018) on the ‘non-disruptive disruptions’ that he argues are at the heart of the ‘new green spirit of capitalism’. Non-disruptive disruptions are ‘technologies that can deliver “solutions” without actually changing much of what causes the underlying problems’ (10). The palatable disruption concept offers a way to critically assess the “ethic of care” that is promoted in a post-milk utopia. Our assessment of the politics of plant milk speaks to efforts to transition to more environmentally sustainable and socially just agri-food systems by responsibilizing consumer-citizens (Lockie 2009; Johnston and Szabo 2011; Roe and Buser 2016). Mylks, we argue, encourage people to rebel just enough to switch from dairy milk to mylk while entreating them to remain devoted consumers of commodity mylk (*and* dairy milk). The post-milk imaginary procures an “unreflexive politics” (c.f. DuPuis and Goodman 2005, p. 361) and neoliberal consumer-subjects by individualizing systemic problems of environment, health, and animal welfare. This serves to bolster corporate knowledge claims about sustainability, entrench market mechanisms, and reify commodity food as a solution.

To critically assess plant milk, we engage with food studies literature on AFNs. This work has demonstrated the challenges of delivering more ethical, environmentally sustainable, healthy, and just food systems through niche production-consumption networks that can exclude producers and consumers (DuPuis and Goodman 2005; Guthman 2008; Alkon and Mares 2012). AFNs are critiqued for the ease with which they are subsumed into productivist logics and coercive politics that undermine the ideals behind food movements (Guthman 2004; Goodman et al. 2012). In particular, we speak to studies that conceptualize consumption as empowering the retail preferences of rational and/or emotional economic actors (Clarke et al. 2007; Swaffield 2016; Evans et al. 2017). We acknowledge work demonstrating that consumption is a complex affective, social and political act, and one that is entangled in networks of concern that stretch well beyond the individual in the here and now (Miller and Rose 1997; Hayes-Conroy 2013, 2010; Carolan 2016). As DuPuis (2000) demonstrates in her work on organic milk, consumers are neither entirely sovereign in making decisions nor entirely victims of marketing. At the same time, we recognize that this green consumerism ‘responsibilizes’ consumers to make environmentally sustainable choices in ways that can entrench the political economic status quo by positioning market exchange as the solution to problems caused by excessive consumption and corollary overproduction (Goodman 2004; Shove 2010; Jones et al. 2011).

Our case study expands on this work by examining how plant-based food futures are shaped, by whom, and to what ends. We describe how palatability is choreographed to secure affective continuity in user experience while conferring an aspirational sense of novelty and disruption. In this way, mylks resemble white middle-class social improvement efforts that constructed dairy milk as a “perfect food” (DuPuis 2002). Promises of perfection, purity, and social change appear in mylk marketing, such as Oatly’s promotion of a post-milk future, which presumes that avoiding milk will rectify issues stemming from agro-industrial dairy production. We suggest that within this post-milk utopia lies a dichotomous ethic of care: an assumption that avoiding dairy can address these issues in the dairy system. This procures an “unreflexive politics” (DuPuis and Goodman 2005, p. 361), privileging food companies to enact a post-milk world. We point to the contradictions and restrictive contingencies that arise in establishing an ethic of care based on such a consumer-company relationship.

## Researching palatability

This paper’s argument derives from an analysis of the existing literatures on the framings of dairy milk, market research information on the mylk sector, the packaging and marketing campaigns of a range of brands, and interviews with 12 representatives of mylk companies, their suppliers, and associations promoting plant-based. We concentrate on the four mylk varieties with the largest market share: almond, soy, coconut, and oat (The Good Food Institute 2019). We selected brands to capture a range of company sizes and histories, including: Alpro and Silk (large companies more than 30 years old); Oatly and Liquats (mid-size companies more than 20 years old); Califia Farms; Plenish Drinks, Rude Health, Rebel Kitchen, and Ripple (smaller companies less than 10 years old).

Our methodology develops critical food studies work on consumer choice, food system transitions, and AFNs, particularly that which focuses on food companies’ ‘mobilization of affective and emotional registers’ (Doyle et al. 2019, p. 3). DuPuis (2000) has analyzed the various cultural framings found on cartons of organic milk and what they say to consumers. Carolan (2015) demonstrates the value of interviewing what he calls ‘the tastemakers’ in food companies and how the food industry aims to activate consumers’ emotional registers in product development and marketing. Sexton et al. (2019) examine how cultured meat is framed with narratives of alternativeness that entice consumers through stories of what is both present and what is absent in the products. This work, in turn, builds on a long history of research in cultural studies that takes marketing as the ‘poetry of capitalism’ (Barthes 1972; Williamson, 1978) and seeks to ‘lay bare the prejudices that lie behind the smooth surface of the visible’ (Rose 2007, p. 76). Such visual methodology requires close critical reading of marketing materials—attending as much to absences as to presences—and awareness of the vital role of intertextuality in creating meaning, cultural value, and emotional experience (Lorimer 2010).

Building on these studies, we take palatability to be a multisensory, affective experience that emerges from both the visual experience of the ‘affective surfaces’ (cf. Forsyth et al. 2013) of milk packaging and marketing, and the taste experience of the mylks themselves. We examine taste and its disruption as both material and semiotic processes (Roe 2006; Hayes-Conroy 2010; Evans and Miele 2012). In keeping with other work in this vein (Longhurst et al. 2008; Mann et al. 2011; Sexton 2016), we tasted the products we describe and explored how the affective experience of drinking mylk is conceived and modified by those in the trade. Informed by thinking in the industry science of behavior change (Marteau 2018), we trace how the claimed disruption of mylk involves both pre-discursive and discursive interventions that work on consumers’ ‘slow’ and ‘fast’ thinking (Kahneman 2011) in ways that far exceed narrow understandings of rational economic action. This element of our methodology is important as mylks have excelled in their ability to create emotional connections through their social media allure and appeal to consumers’ palates and habits (Levitt 2018).

The paper starts with a discussion of the historical narratives surrounding dairy milk to establish how plant mylks inherit framings of milk as: (i) pure, wholesome, and healthy; (ii) tasty and convenient; (iii) risky and environmentally damaging; and (iv) cruel and inhumane. We then explore how mylks navigate these framings to remake the milk experience as palatable and disruptive. Our analysis illustrates three techniques of palatable disruption, documenting how plant mylks: (i) ‘taste good’ by securing affective continuity in taste experience; (ii) ‘feel good’ by affirming and facilitating broadcast (i.e. virtue signaling) of one’s environmental and health values while avoiding unpalatable political registers of disgust and agonism; and thereby (iii) maintain the political and cultural economic status quo through the consumption of agro-industrial food. In conclusion, we identify the political economic characteristics and implications of this model of change in the food system, which we flag as priorities for future research.

## The discursive landscape of dairy milk

Contemporary forms of commodity milk production-consumption are the result of discursive and material processes; the work of innumerable actors and institutions (Smith-Howard 2014). We focus on the discursive constructions of milk, which are entangled with political economies and ecologies of production (DuPuis 2002). While milk’s cultural, political, and economic importance spans the world (Valenze 2012; Wiley 2008), this article concentrates on the US and UK. Over the past 200 years, milk has been continuously re-framed in response to changing societal values about food, animals, and the environment (DuPuis 2002; Freidberg 2009; Atkins 2010). Claims about milk’s healthiness, ethics, wholesomeness, and worth have been repeatedly contested and various ‘better’ alternative dairy production-consumption practices have emerged to counter skepticism. Here we briefly outline the framings of milk that mylk producers in North America and the UK inherit, navigate and repurpose to justify their products’ alterity. Drawing in part on existing literatures on milk’s cultural and political history (e.g. DuPuis 2002; Freidberg 2009; Atkins 2010; Valenze 2012; Wiley 2014), we suggest that mylks curate affirmative cultural signifiers of milk’s palatability as: (i) wholesome and healthy; and (ii) tasty and convenient. But mylks must also depart from the framings that make dairy disgusting, in which: (iii) milk is risky and environmentally destructive; and (iv) milk is murder.

### Milk as wholesome and healthy

Humans have relied on animal milk as a source of calories and nutrients in many regions of the world for 3000 to 7000 years (Salque et al. 2013). Milk is the only food that human bodies are also capable of producing to feed offspring, and animal milk has frequently been associated with motherhood, vitality, and the sacred (DuPuis 2002; Valenze 2012; Wiley 2014). Its dietary importance among some societies generated reverence for milk, the animals producing it, and the people tending to them; a mythical status that is captured in art and literature (Kurlansky 2018). In places where a majority of people could digest lactose, dairy milk was perhaps seen as the original ‘superfood’. In medieval England, dairy was a crucial food source for the rural poor, for whom it was regarded as ‘white meat’ (Freidberg 2009). The socially-constructed image of milk as wholesome and pastoral amongst urban, industrial consumers is a more recent abstraction, yet one that mobilizes similar narratives of health and motherhood towards milk’s commodification (DuPuis 2002; Wiley 2014).

Historians suggest that this imaginary took hold once urban consumers were alienated from relations of production. The framing of milk as a nourishing, healthy food for urban citizens dovetailed with the separation of cities from rural areas, the rise of refrigerated train car technology, and laws that favored larger dairies that had the ability to pasteurize at scale (Freidberg 2009; Atkins 2010). Milk marketing campaigns have repeatedly developed milk’s wholesome palatability through use of the nostalgic conception of the pastoral (Marx 2000; Wiley 2008; Paxson 2013). These positive traits persist to this day in the advertising of dairy milk, particularly in its organic variant (DuPuis 2000) as well as almond milk (Bladow 2015). This affirmative connotation of milk has overlapped with a parallel discourse of milk as nutritious, which has repeatedly targeted ‘weak’ women and infants—promoting the substitution of breast milk for cow’s milk (Atkins 2010; Dupuis 2002; Valenze 2012). Latterly—for example in the high-profile US ‘got milk’ campaign—this gave way to a more general mobilization of the archetypal healthy bodies of celebrity sportsmen and women.

### Milk as tasty, affordable, and convenient

Milk’s commodification in twentieth century North America and Europe was entangled with a story of progress that centered on the modernization of production processes to increase the availability of pasteurized milk and to ensure healthy, strong bodies. Milk’s idealized position as a ‘perfect food’ (DuPuis 2002) merged with a narrative of modern nation building through increasing production and reliable year-round supply. These were enabled by transportation networks that made milk good value in terms of price to nutrition (DuPuis 2002; Freidberg 2009). Cheap commodity milk was upheld through government subsidy structures that furthered dairy farm specialization. These political economic and cultural processes continued to fuse with the imaginary of milk as wholesome and pure and were central to milk becoming a household staple in the US and UK following the second World War (Valenze 2012). Surplus milk and corn were conjoined in the marketing of the cereal breakfast that became a US institution (Kellogg’s Corn Flakes being a prime example). Standardizing the lipid, protein, and sugar content was a key aspect of milk’s imaginary as modern (Atkins 2010). In the twenty-first century, milk processing and distribution were even more heavily standardized to ensure consistent taste and reliable food safety (Smith-Howard 2014). This standardization, together with an emphasis on reducing cost through larger farms and the homogenization of cattle genetic diversity and feed sources, led to a toning down of the intensity and diversity of milk flavors (Freidberg 2009; Levitt 2018). Tasty milk is promoted more through the absence of the flavors associated with fermentation, which might indicate spoiling and the possibility of food poisoning, in marked contrast to the promotion of cheese (Paxson 2013). The convenient qualities of milk are characterized more by a consistent ‘mouth-feel’ and dependable material performance in bowl, pot and cup. The taste of this bulk milk can then be enhanced, and economic value added, through a proliferating range of added flavors and forms of ‘fortification’.

### Milk as risky and environmentally damaging

As milk consumption in urban areas grew at the start of the twentieth century, so did the distance that milk travelled, leading to increased risk of milk spoiling and of potentially fatal disease.^3^ Some urban consumers were skeptical of milk coming from outside the city, which could go off or be skimmed or adulterated by unscrupulous merchants. These practices were widespread in the UK and US (Freidberg 2009; Atkins 2010). This established a framing of milk as risky and unhealthy, which has been reinvigorated as a result of more recent public health research establishing links between cardiovascular disease and the consumption of saturated fats.^4^ By the end of the twentieth century, a perception had emerged in some circles that milk and dairy products were unhealthy (Valenze 2012). Meanwhile, concerns in the US about the use of antibiotics and growth hormones in milk production led to renewed disquiet about the purity of milk and drove demand for organic milk (DuPuis 2000). Over the past decade, there has been increasing anxiety about the negative environmental impacts of dairy production. These concerns initially centered on the impacts on water, land use, and biodiversity caused by intensive dairy systems and have since expanded to focus on the greenhouse gas emissions of industrial dairy production (FAO 2006; Foote et al. 2015; Springmann et al. 2016; Poore and Nemececk 2018). Prominent examples of this negative framing include campaigning films like *Cowspiracy* (Andersen and Kuhn 2014) and *What the Health* (Kuhn et al. 2017) that make visible the health impacts and ecological relations of meat and dairy to create doubt and unease amongst consumers.

### Milk as inhumane

Concerns about animal welfare in dairy production systems can be traced back to at least the nineteenth century (Fisher 2018). In the USA, images and descriptions of urban ‘swill dairies’ (which fed cows mainly with by-products of beer brewing) circulated in newspapers in Boston and New York. These exposés led to public outcry, government regulations, and eventually the closure of urban dairies (Freidberg 2009). In the twentieth and twenty-first centuries, animal welfare and animal rights campaigns have persistently criticized the dairy industry for animal abuse. Led by non-governmental organizations such as People for the Ethical Treatment of Animals (PETA) and the Vegan Society, such campaigns have engendered a strongly negative view of dairy’s impacts on animal welfare (Mylan et al. 2018). This negative perception further established itself in the public consciousness with the use of high-profile advertising, demonstrations, social media, and an accelerated film campaign with titles such as *Food, Inc*. (Kenner et al. 2008) and *Eating Animals* (Foer et al. 2017). These framings tend to accentuate the corporeal affinities between human and bovine bodies and the physical and emotional violence associated with the dairy industry (Tulloch and Judge 2018). They reference the severed maternal-infant bond (‘not your mom, not your milk’) and use shocking images to present ‘milk as murder’. In so doing they engender disgust, subverting the more traditional perceptions of the wholesome palatability of milk.

## Framing plant mylks

Over the past few decades, campaigns for or against milk have strategically mobilized these conceptualizations in ways that shape and connect with consumers’ emotions. It is from this contested melee of arguments and feelings that the current framings of plant mylks have emerged. In taking their products into the mainstream, mylk companies must navigate a contentious material and semiotic terrain to curate the palatability of milk, while also promising to disrupt the status quo to address consumers’ concerns. We focus our analysis on three prominent framings.

### Looks, acts, and tastes like dairy milk

*‘*When should you use it? Whenever you would use old-school *milk* from cows—chilled in a glass, for cooking or baking—in exactly the same amounts.’ (Oatly 2019, emphasis in original).

Perhaps the most obvious feature of the mylk companies’ efforts to maintain palatability is the ways in which they strive to mimic how dairy milk looks, acts and tastes. Mylks exist invariably as white ‘milky’ liquids, and plants are selected and processed with this end in mind. Good Hemp Barista Seed Milk, for example, claims on the carton that it is ‘naturally white.’ Three Ones Almond Milk notes on the carton that it is ‘pure white.’ Imagery of milky liquid feature prominently on packaging. Sometimes, as in the Plenish advertisement in Fig. 1, milk is exploding or otherwise emanating from almonds, soybeans or other plant components. In the case of the less self-consciously ‘radical’ mylk brands, such as Alpro and Silk, advertisements depict a pitcher of creamy plant milk pouring into a bowl filled with breakfast cereal. The quintessential modern Western breakfast.

**Fig. 3:**
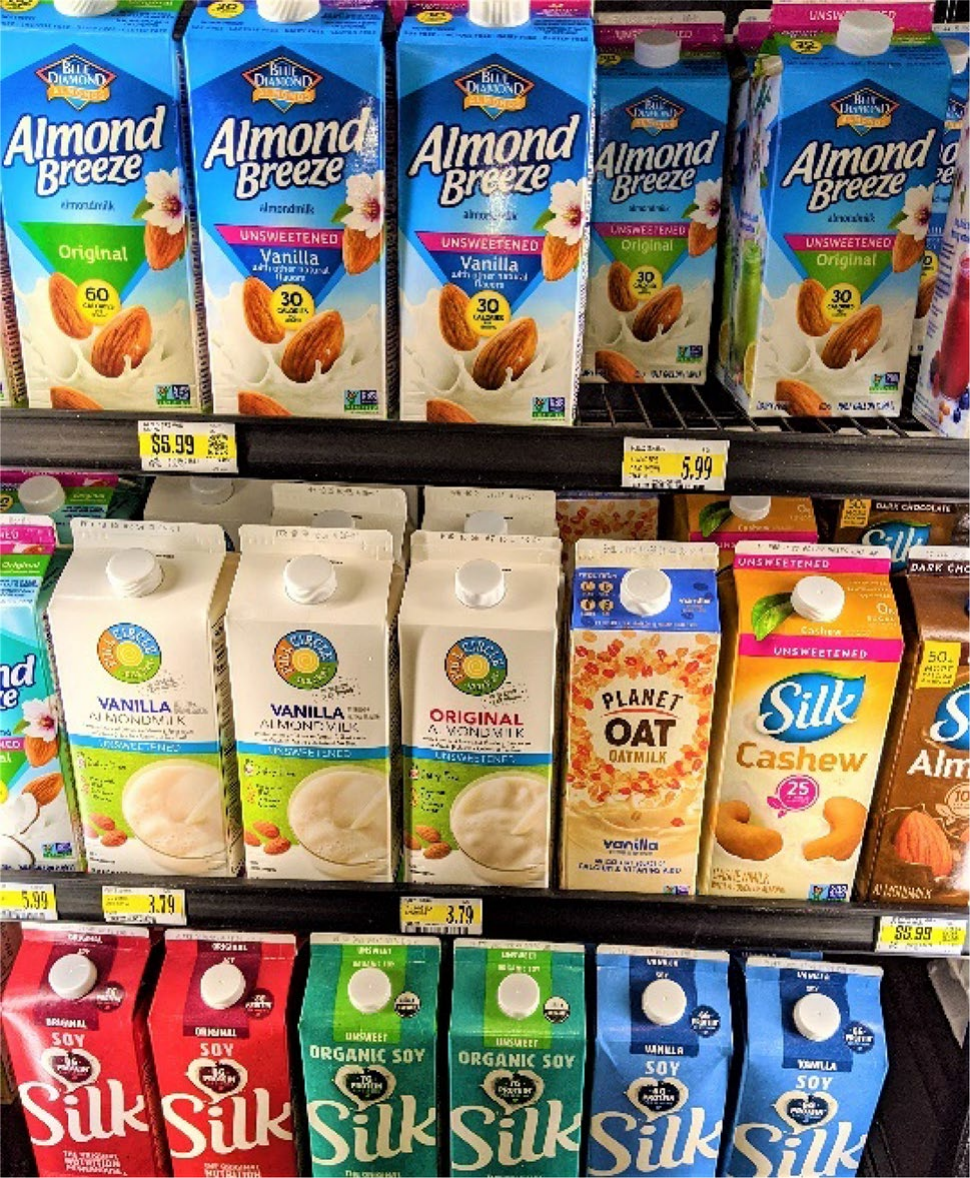
Refrigerated mylk in supermarket. Photo by Nathan Clay, 2020

When it comes to taste, some mylks (such as Rude Health and Plenish) claim to replicate the refreshing, ‘pure’ taste of (chilled) dairy milk, and to derive this purity from using only few ingredients. Many brands include flavorings, stabilizers, emulsifiers, and refined sugars in an effort to mimic the texture or mouth feel of milk. A carton of Rebel Kitchen Mylk exemplifies this, claiming ‘what we all really want from a dairy free alternative is that it tastes & looks just like real milk. Right?’ In trends comparable with dairy milk, many mylks are also flavored, most often with vanilla, in ways that explicitly depart from the claimed blandness of pure milk. As we examine in detail below, the form and style of the packaging often purposively resembles that of commodity dairy milk. Mylks are also sold in ways that resemble dairy milk, located in refrigerated aisles adjacent to dairy milks (Fig. 3) or next to the UHT milks in the ambient aisles. Likewise, in coffee shops, which have proven to be crucial spaces in which consumers first try mylk, they are sold with a promise of comparable or even enhanced frothability. Several industry respondents emphasized that it is these ‘flavor cues’ that drive mylk consumers far more than environment or health claims.^5^

### Nutritious, powerful, pure

“This is THE almond milk YOU DESERVE” (Califia Farms 2019).

**Fig. 4:**
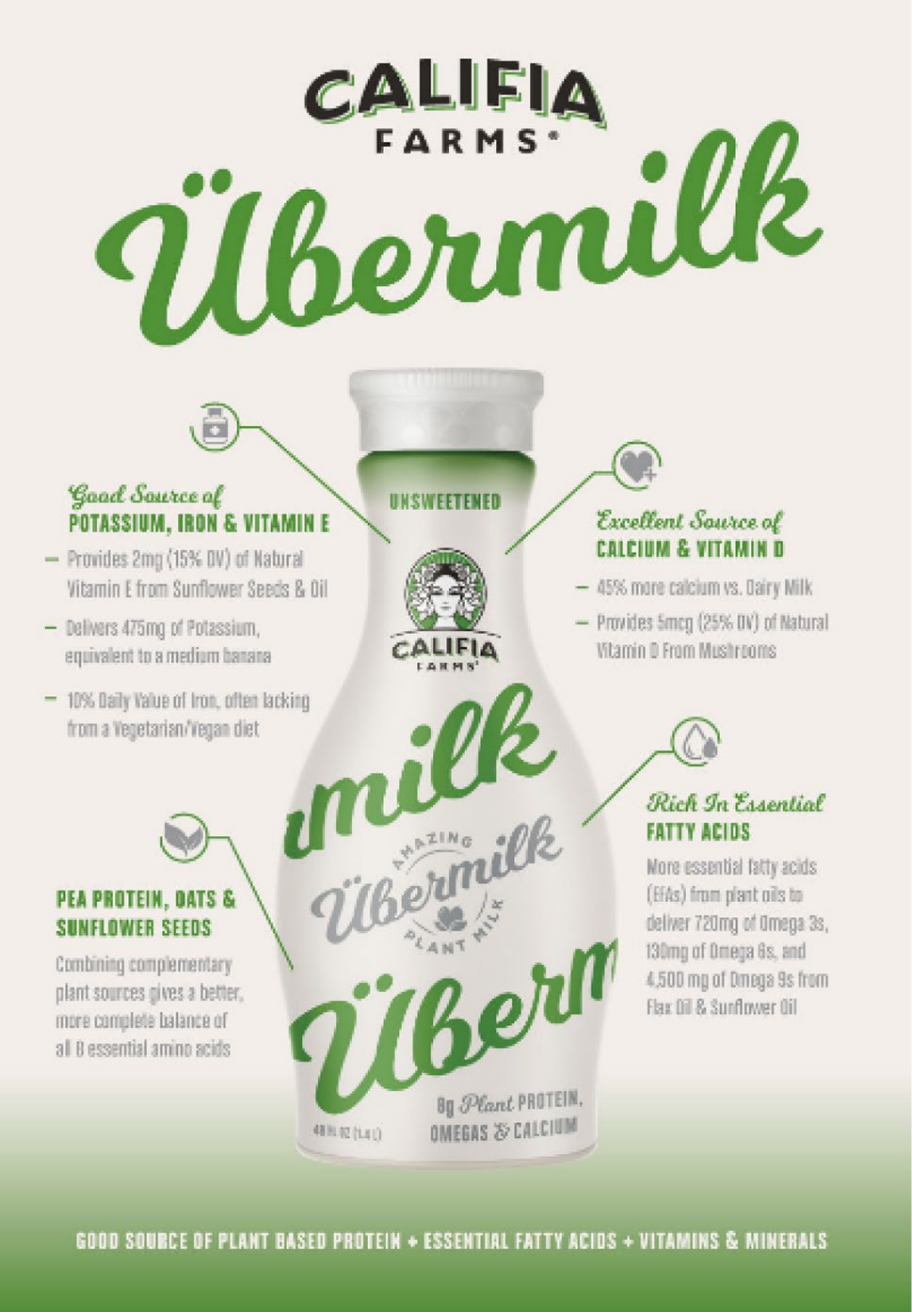
Califia Farms Ubermilk

“Plant powered” is a ubiquitous phrase among mylks. It harnesses preexisting understandings of the health benefits of protein while overcoming the potential harms of dairy milk. Mylks’ wellness claims span myriad definitions of health and multiple epistemologies through which it might be known and achieved. Many brands reference nutrition, claiming to maintain or even enhance milk’s calcium and protein, although this is inevitably through the addition of supplements and patented processes of protein extraction. Califia Farms Ubermilk, for example, claims 45% more calcium than dairy milk and a range of other essential nutrients (Fig. 4). Some brands pick up on concerns over heart health and dairy fats. Hearts (a regulated symbol of the American Heart Association) adorn PepsiCo’s Quaker Oat Beverage. A Plenish Drinks mylk carton says ‘When you replace saturated fats with heart-healthy monounsaturated fats found in this hazelnut m*lk, you can reduce blood cholesterol levels. High cholesterol can lead to heart disease, so make the switch for good’.

The Plenish package goes on to claim that ‘by proactively filling up on natural, healthy ingredients and harnessing the mighty power of plants, you can press on and crush it!’ This appeal channels the current infatuation with protein and a prevalent mantra of overcoming challenges of ordinary life through food.^6^ Indeed, many mylks leverage a generic image of strength and power. Slogans such Alpro’s ‘enjoy plant power’ convey an image that bodily strength is possible through plants, a discourse which resonates with the highly physical, masculinist vegan identity that is coming to prominence amongst some high-profile vegan advocates (Sexton et al. 2019). Here mylks eschew past gendering of milk as female while amplifying earlier framings that associate dairy milk with sporting prowess and the bodies of celebrities known for their physique.

Many mylks are marketed with more diffuse notions of health, likely because mylks lack the quantity and variety of nutrients found in dairy milk. These abstract health claims are captured in words like ‘wellness’ and ‘cleanliness’, as well as by Instagram-friendly iconography. Such promises of holistic health are epitomized by slogans like ‘eat right, stay brilliant’ (Rude Health) and ‘feel good food’ (Happy Planet Oatmilk). As a signifier of wellness, Mylks frequently gesture to what is absent. Often this is dairy. For example, Rebel Kitchen says of its semi-skimmed mylk: ‘why the y? It’s made from plants, not cows.’ Notions of purity similarly abound. As Rude Health Ultimate Almond states, ‘we only use the kinds of ingredients you’d have in your own kitchen—nothing artificial, nothing refined. We source our ingredients from fields, orchards and vines—not laboratories.’ This dovetails with the health as purity discourse that underlies the recent ‘clean eating’ trend. More overtly, mylk packaging frequently states what is absent in terms of calories and added sugar.

### Green and compassionate

“We make compassionate food for passionate people” (So Delicious CoconutMilk).

Certifications abound on mylk cartons. ‘Vegan’ is ubiquitous. ‘GMO-free’ adorns most mylk cartons in the US. A vital storyline across brands is the lower environmental footprint of plant milks relative to dairy milk. Yet this is displayed to different degrees. Dairy-owned brands like Alpro appear more reserved, relegating discussion of environmental footprint to a small text box on the side of the carton. Others are louder. Plenish challenges: ‘if you want to change the world change your milk.’ Industry respondents informed us that environmental claims are regulated far less than are health claims. Many brands use relatively vague imagery to convey sustainability. For example, Milkadamia states on the side of the carton that it uses ‘free range trees, trees supporting life, not trees on life support... in total harmony with the earth, nurtured by natural rainfall and sunshine.’ Others display environmental footprints based on product life cycle assessments. Oatly displays its oatmilk’s carbon footprint on each container. Plenish Drinks has an environmental footprint calculator on its website.

Some European almond and soymilk brands address environmental concerns through the identification of geographic origin. For example, Provamel Almond [beverage] has a map of the world with an arrow pointing to Europe alongside the text: ‘We care about where we source our ingredients. That’s our promise to you.’ Such coding tacitly acknowledges, while also distancing from, the controversial intensive almond supply chains that are booming in drought-prone California.^7^ For reasons that we go into below, mylk brands devote little explicit attention to animal welfare in their marketing. A few, such as Good Karma (owned by US dairy corporation Dean Foods), make implicit reference to ethics, but the majority choose to emphasize their ‘plant-based’ rather than ‘animal-free’ constitution. They leave animals—with their powerful affective associations with cruelty and disgust—absent and unsaid.

To summarize, through this work plant milk companies successfully inherit framings of milk as wholesome and convenient, while circumventing framings of milk as cruel and environmentally damaging. Some frame their products as the refined continuation of milk tradition, while others present a disruptive break with an anachronistic dairy past and a step toward a post-milk future.

## Palatable disruption

“Good for you products that are also good for the planet” (Califia Farms).

In this section, we explore the politics currently afforded by mylk. We present the mainstreaming of plant-based dairy as an example of Goldstein’s non-disruptive disruptions—in which grandiose claims of challenging an environmentally damaging status quo provide ‘moral legitimacy and affective force for proposals to irrevocably transform capitalism into a more environmentally virtuous economy; still capitalism just a better, greener version’ (2018, p. 30). We advance this assertion and its relevance to food by developing the concept of palatable disruption. A palatable disruption is a widely promulgated claim for a change in the food system that: (i) maintains continuity in taste experience; (ii) performs a politics that feels good to citizen-consumers; and (iii) works to sustain or even amplify elements of the political economic status quo that are palatable to corporate interests. We explore these three themes below with attention to how flexitarian consumer-subjects are produced through neoliberal mechanisms that underwrite the palatable disruption of mylk. The case of mylk provides a window onto the broader trend of plant-based foods.

### Consumption continuity

Considering all the talk of disruption, it is perhaps striking how much mylks look and taste like milk. Yet, it is this interchangeability that has made mylks such effective commodities. As we demonstrated above, mylk companies have worked to secure continuity in their users’ experience, even as they shift mylk’s material composition and herald its disruptive potential. There is no necessary reason why mylk should be white or served cold. Liquid plant products don’t need to be used to dilute coffee, bulk out smoothies, or moisten cereals. But this is how and where they invariably end up. As such, they testify to an inertia in the North America and European food system; a cultural economy of western breakfast that resists transformation. Like Goldstein’s (2018) Cleantech entrepreneurs, mylk companies ultimately seek marginal gains in established markets for commodity accompaniments to cereal and coffee. By the time mylks get to the coffee shop or supermarket shelves, these products are not intended to shift ingrained habits to create new markets. Companies are aware of how hard it is to get consumers to try new products, and instead seek to replicate the palatable experience of milk consumption.

**Fig. 5:**
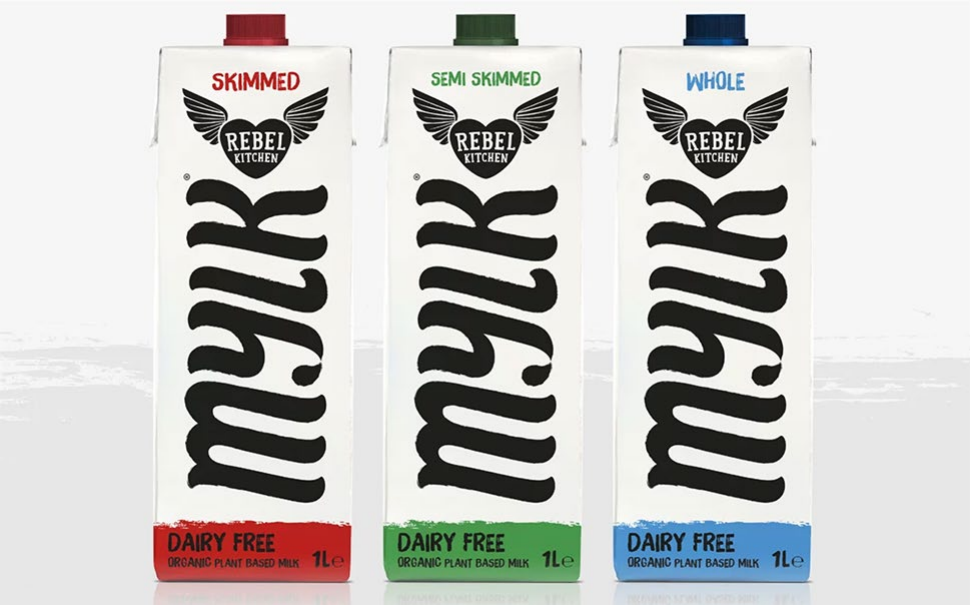
Rebel kitchen cartons

This continuity can be understood by attending to how the practical and affective dimensions of the mylk consumption experience are choreographed by the applied sciences of food product formulation, packaging, and retail. As critics have observed, these are established knowledge practices that have mastered how to create, shape, and ultimately govern consumer desires, often through techniques that work more on bodily feelings and habits than through appeals to rational choice (Moss 2013; Carolan 2015; Schatzker 2015). Making mylks palatable involves drawing on gastronomic science and the technical skills of food processing to reformulate their taste away from the ‘mealy’ and ‘beany’ flavor of earlier plant mylks that catered to vegans and the lactose intolerant. Mylks have been smoothened, sweetened and refined to match the taste and mouth feel of dairy. The carton of Rebel Kitchen Mylk that we encountered above, which claims that ‘what we all really want from a dairy free alternative is that it tastes & looks just like real milk’, goes on to note the various tastes of milk which its team of taste profilers sought to emulate with plant-based ingredients. In response to the rhetorical question ‘how do we make it so mylky?’ the package lists ‘coconut cream for creaminess... brown rice for sweetness... cashew for earthiness... nutritional yeast for grassiness’. This strategy also speaks to a marketing trend of incorporating ‘tasting notes’ on various foods, a practice imported from wine and designed to flatter consumers for their appreciation of flavor subtleties. Some brands, such as Oatly and Rebel Kitchen, offer ‘skimmed’ and ‘whole fat’ versions, in the latter of which fats are added to mylks (Fig. 5). This process is reminiscent of industrial dairy milk production, which skims all milk and adds fat back as needed.

These techniques often center on producing sweetness in mylks to resemble the lactose flavor of dairy milk. Often, sweetness is produced through adding refined sugars. Many mylk brands offer unsweetened versions, which claim to include no added sugar. Yet, these claims may be misleading. Oatly’s oat milk, for example, relies on enzymes to break down plant starch into simple sugars. Oatly recently stopped advertising no added sugars following a complaint by rival Campbell Foods, whose oatmilk (sold through their Pacific Foods subsidiary) contains 17 g of sugar per serving (Watson 2019). Mylks also seek to replicate the frothability of dairy milk that is so valued in contemporary urban coffee culture. Oatly pioneered this with their ‘barista’ edition. Many other companies have since followed, blending mylks with plant oils as well as acidity regulators that suppress separation once coffee is added. For example, a mylk company representative explained how brand loyalty was built through this consistent re-creation of coffeeshop rituals: foaming to the right consistency, mixing with coffee at the right ratio without separating, and offering the capacity for latte art. He explained how the consumers’ affective experience of their product is preeminent, and it is only later that a story of health or environmental sustainability comes into play. Haptic and olfactory consistency may trump ethical exhortation as a driver of mylk sales.

This attention to embodied practice and affective experience also informs the science of product location and the choreography of the supermarket shopping experience. Ethnographic studies on supermarket design and use have revealed the subtle and sophisticated ways in which consumers are trained and habituated to navigate grocery stores and fill their baskets (Colls and Evans 2008; Johnston and Szabo 2011; Carolan 2018). A growth-oriented corporate mindset often underlies these supermarket strategies, even among ‘alternative’ outlets such as Whole Foods Market, where retail spaces are imbued with feminized notions of care through food (Johnston 2008; Cairns and Johnston 2015). This work suggests that much consumer ‘choice’ is habituated and subconscious, and that consumption acts are choreographed to limit conscious decision making (Sexton 2018). Food companies pay a premium to have their products placed at the end of aisles (or ‘endcaps’) and at desired heights on shelves. These premium locations are understood to matter as much as price and special offers in driving sales. Dairy milk is commonly a loss-leading staple in supermarkets and its location is carefully planned: far enough from the entrance that consumers must pass other tempting aisles to reach it, but not so far or so hidden as to be inconvenient.

In contrast, plant mylks have historically been found in peripheral ‘alternative’ or health food aisles where there is limited chance of serendipitous encounter. To normalize their brands, some mylk companies have paid a premium to have their products located in the refrigerated dairy aisle. To enhance this affective continuity in shopping experience, in 2016 Tesco and Alpro teamed up to sell their milk chilled, although this is not required for food safety (White 2016). The US companies Silk and Almond Breeze similarly relied on connections with prominent dairy milk companies to gain access to privileged refrigerated shelf-space (Franklin-Wallis 2019). Companies like Rebel Kitchen have even sought to emulate the packaging of dairy milk, using rectangular cartons with caricatured bovine white and black text, and a familiar range of single color tones (red, green, blue) to denote to UK consumers skimmed, semi-skimmed, or whole mylk options (Fig. 5).

The commercial success of this making palatable is evidenced in both the quantity of sales and the crossover between consumers of both dairy and plant milks. Market research suggests that consumers’ adoption of mylks has not involved the like for like replacement of dairy products. Around 80% of households that purchase mylks also buy dairy milk (Mintel 2019). This fact is not lost on mylk companies, who target flexitarian consumers rather than vegans. Interview participants at one mylk company noted market research that plant-based mylk consumers actually consume *more* dairy milk. The reason given was that mylk drinkers tend to be ‘foodies,’ that is, people who are more interested in food in general. Despite claims of a post-milk generation, for the time being at least it appears that the rise of plant mylks represents a net increase in the consumption of packaged white liquid drinks. This would cast doubts on mylk companies’ claims for environmental sustainability through the consumption of ‘less’ milk.

### Cozy politics for flexitarians

This choreography of the mylk experience is given meaning through the range of storytelling practices we encountered above. Together these interventions work to create, shape and subjectify a ‘good consumer’. Advertising has long been invested in reflecting and morphing modes of social distinction, channeling cultural identities to create affirmative product associations. The framings of milk and its alternatives are animated through a range of affective styles.^8^ Advertisements reflect, refract, and sometimes forge social norms and identities. For example, we have seen in the images above how the framing of milk as wholesome is enabled by an affective style that conjoins rural iconography of white bodies, traditional technology, and sunlit landscapes with retro visual filters, pastoral music, and linear editing. The result is a nostalgic sense of continued social order. In contrast, framings of milk as murder feature animal head shots and industrial technology conjoined with guttural animal sounds. This harsh soundscape, frequent jump cuts, and shaky low-fi image quality suggests covert provenance and shocks viewers, who are disgusted with the palpable sense of social disorder.

Dairy-owned mylk brands, like Alpro, have chosen to persevere with the nostalgic pastoral style to promote plant milks as the healthy continuation of wholesome animal milk. We are to believe that there is nothing radical about their products; that they offer a logical technological innovation that replaces cows with plants. The tenor of this *cozy marketing* is exemplified by the absence of reference to animals and animal welfare. These mylk brands calculate that long-standing vegan consumers do not need reminding of this, while new flexitarian consumers prefer affirmative connections with ideals such as cleanliness, power, and wellness. Mylk companies do not want to invoke powerful gut feelings of disgust at animal suffering, even if no animals are harmed in plant mylk production. Our interviewees at the more ostensibly disruptive brands expressed reservations about referencing animals due to the risk of alienating the 98% of their consumers that are not vegan. Mylks thus inherit and benefit from the unpalatable framings of milk offered by campaigning vegan and animal rights organizations, without needing to give them explicit publicity: they are compassionate by default. Refraining from revolting and shaming consumers is especially important given that the vast majority of mylk drinkers also consume dairy. Even the most overt mention of animals (such as Oatly’s slogan ‘wow, no cow!’) are not explicitly related to animal rights but rather to the absence of animals, or animals as ‘non-stuff’ (Sexton 2016).

The vegan studies scholar Richard White argues that this rise of the ‘vegan-consumer’ and the flexitarian food subject represents a radical departure from ‘vegan-activism’. The latter is commonly associated with abstinence, a withdrawal from mainstream food cultures, and an antagonistic politics of protest. Veganism was commonly sidelined by mainstream media as extremist (Cole and Morgan 2011). In contrast, most mylks are promoted as ‘plant-based’ or ‘plant-forward’ rather than animal-free. This offers a seemingly cozy, harmless and aspirational coding for mylk consumption, untainted by associations with ‘reactionary’ animal rights movements (White 2018; Davis Undated).

Those promoting mylk as a radical break from animal milk develop a different, affirmative style of disruption: their mylks are neither cozy, nor revolting, but revolutionary. One tactic is to channel the longer history in advertising of building product associations with the celebrities, music, fashion and iconography of youthful rebellion. Twentieth century shifts in social values like the hippy, rock or punk movements have long been deployed by advertisers seeking to differentiate their products away from mass marketing and towards lifestyle marketing to rebuild trust through co-opting elements of counterculture (Binkley 2003). Today, mylks deploy self-aware advertising to reach a millennial generation that is not only skeptical of corporate power but also adept at decoding and dismissing traditional advertising. Campaigns have shifted from celebrity endorsement to relying on social media and the established advertising tactic of irony, playing with intertextuality in their images and discourse to acknowledge their viewers’ cultural sophistication (Jackson and Taylor 1996) and speak to their multiple identities as both citizens and consumers.

Arguably the most effective tactic used by disruptive brands to tap into the millennial zeitgeist to drive sales has been to combine irony with transparency in effort to build a more authentic, trusting relationship with consumers. Oatly’s Creative Director John Schoolcraft captures this sentiment in describing Oatly as a ‘challenger brand’:

Being a challenger is having a mindset of realizing you're trying to change something, rather than be a challenger to be cool and help sell more product. Because consumers will be able to feel it. Of course we want to sell our product, but we want to challenge the norms at the same time, and that’s bigger. If you can get that right, you’re going to sell a lot of product, and we need to sell product so that can continue to do what we’re doing. (The Challenger Project 2016).

Michael Lee, the strategic director for international markets at Oatly, notes that this type of branding requires both an ironic ‘Oatly tone of voice’ that ‘flexes on the nonsensical’ as ‘it just becomes lame when you start preaching it in your communications’, but it also requires a veneer of transparency to acknowledge consumers’ distrust of advertising (Rogers 2019). Advertising commentator Jamie Williams (2019) explains how Oatly has pioneered a tactic known as ‘unadvertising’, which mocks the traditional advertising formula. This style takes the intertextuality of ironic advertising to another level, repurposing the subversive tactics of the 1990s anti-capitalist Adbuster movement in an effort to overcome widespread cynicism about the social role of advertising (Lasn 1999).

**Fig. 6:**
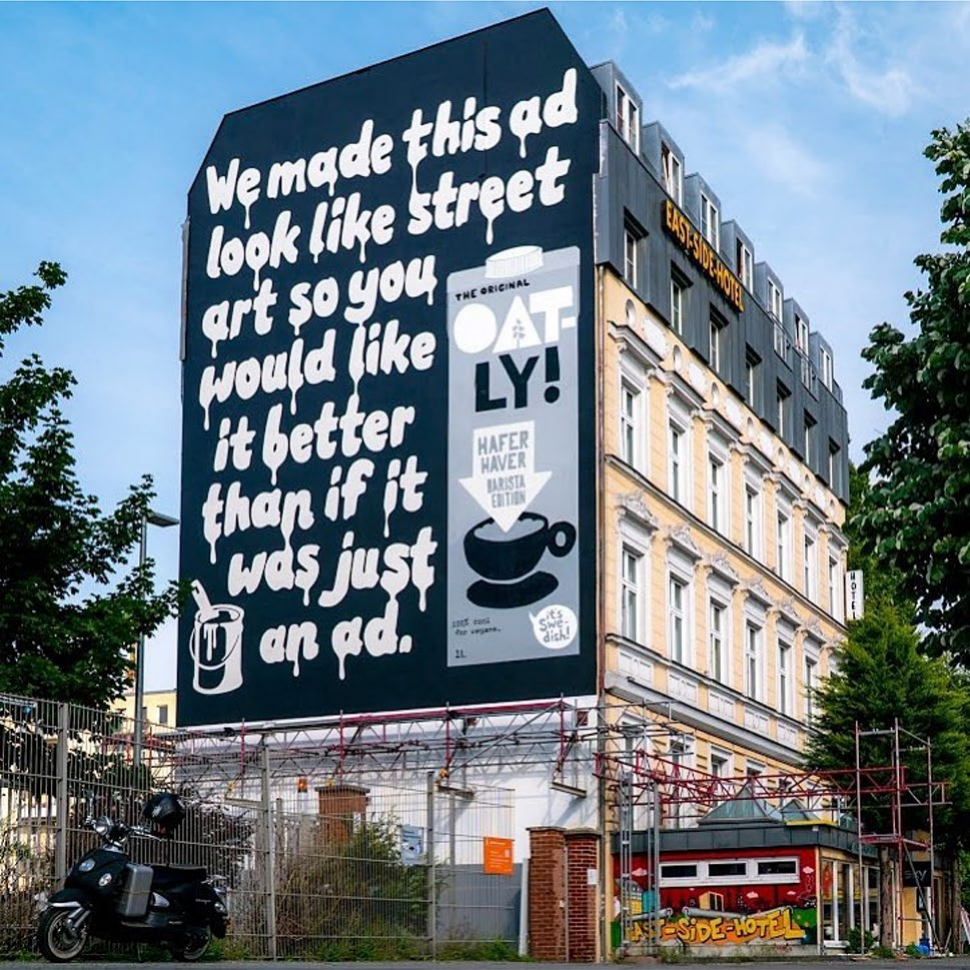
Oatly billboards

Unadvertising is compelling because it makes manifest the ubiquity of advertising, while celebrating the individual ability of millennials to deconstruct and reflect on their own subjectification (Fig. 6). But the disruptive power of this style is ultimately limited by the absences it is willing to make present and the cozy types of affect it finds palatable. As a result, the palatable coziness or bounded self-reflexive edginess of this mode of consumer-led disruption lacks the affective agonism that political theorists hold to be central to the successful functioning of democracy.^9^

The palatable, ‘feel good’ food politics of disruptive mylk advertising—that eschews disgust at animal death and the ‘extremism’ of vegan activism—also evades the disagreeable opinions of those who stand to lose out from this reorganization of the dairy system. It avoids unpalatable ruminations over whose economic interests it serves, and the social relations involved in producing almonds, soy and oats, for example. It certainly can’t stomach questioning the claim that buying more will save the world. In short, there is little space for debate here, in spite of the proliferation of rhetorical ethical questions. While this criticism no doubt sets the bar too high for what we might realistically expect of fast-moving consumer goods, it does allow us to dispel the more outlandish claims that these products will necessarily catalyze political economic disruption.

### The spectacle of care

The careful choreography and sophisticated marketing of the mylk experience is geared towards the creation, subjectification and governance of a set of ‘good’ or ‘ethical’ consumers. In AFNs such as local food (DuPuis and Goodman 2005), organic (Guthman 2004), and fair trade (Goodman 2004), consumers are responsibilized through concepts such as food miles or through third-party certification, underlying which are often relatively rigid norms and imaginaries that can exclude as much as they include. In this final analytical section, we explore the “normative pre-set ‘standards’” (DuPuis and Goodman 2005) that arise through the utopian model of plant mylks. What consumer subjects are required to make plant mylks palatable to the ‘new green spirit of capitalism’ (c.f. Goldstein 2018)? What models of production do these standards promote?

Ethical food consumerism has a checkered history that is well reported in the academic literature. As discussed in the introduction, AFNs emerged by drawing attention to the social, animal and environmental harms caused by agro-industrial systems. Through organic or fair-trade certification, or through community supported agriculture, they enable consumers to ‘vote with their wallets’ and support alternative social-environmental ideals that better align with their values. Studies have traced these food movements’ complicated relationship with ‘conventional’ food systems. AFNs have often been subsumed within a model of neoliberal agro-capitalism which places the consumer as the sovereign political agent in determining how, what, and where food is grown, distributed, and consumed (Goodman et al. 2010, 2012; Guthman 2011; Alkon and Mares 2012). The manifold imperfections of AFNs are seen to boil down to inequalities in ‘who gets to the table’ to eat ‘good’ food and to make political decisions and whether food movements absolve the state of responsibilities to ensure healthy food and environments (Guthman 2008; Hinrichs and Allen 2008; McMahon 2011; Johnston 2017).

The story of mylk as a palatable disruption allows us to develop two strands of this literature. For one, the centrality afforded to the consumer-citizen as the locus of change in the politics of palatable disruption relegates other models of food system transformation that might address more systemic issues. But even more importantly, mylks foster a reliance upon food companies as ethical food system actors. Mylk companies’ promises of disruption hinge on establishing their legitimacy as conduits of food system change and as custodians of a diverse range of consumers’ cares. In short, it is up to the companies whether they adopt practices that stimulate changes in supply chains and yield benefits to social, environmental, and animal welfare dimensions of production.

Some companies go as far as to present themselves as social movements, endeavoring to replicate the sociology of their non-profit precursors. This trend is shown in Oatly’s efforts to forge a ‘post milk generation’. Mylk consumers are invited to imagine themselves as part of a radical social movement, united by their demographic (aspirationally coded as young and enlightened) and counter-posed to an older section of the population (coded as conservative, ignorant and/or reckless). Identification with this ‘neo-tribe’ (Maffesoli 1995), attached to a generational divide, serves to solidify the bond between consumer and company. This is enhanced by the provision of branded goods (t-shirts, loyalty cards, stickers), the creation of social media communities (blog posts, giveaways, and Instagram friendly imagery), and visibility at music festivals and other archetypal generational rights-of-passage events (Rogers 2019). Oatly implores consumers to invest in this relationship—and therefore a collective future—by drinking their milk:

You are one of us now. You are now part of a growing group of people that understand the benefits of eating and drinking plants so your body feels good and so the planet can better cope with the impact we humans place on it (Oatly 2019).

In so doing these brands leverage the radical history of ‘new social movements’ and their politics—including those that sought change through alternative consumption—while sterilizing their potential for democratic transformation. Indeed, disparaging (often older) critics take issue with the ways in which such brands co-opt activist-inspired discourse to stimulate a feeling of urgency and to cultivate the sense of a collective agenda (White 2018). They variously dismiss this social movement simulation as feel-good, techno-optimistic ‘slacktivism’ that helps further entrench the fundamentally neoliberal project of ethical consumption by attaching a revolutionary air to it (Morozov 2013; Dennis 2018). Such consumers stand accused of narcissistic, ‘virtue signaling,’ that is, posting images of their consumption choices to social media in order to depict themselves as ethical. Indeed, social media has been crucial to inscribing the alternativeness of mylks in the collective consciousness. Unlike AFNs, mylk’s alternativeness centers not on networks with distant producers and landscapes but on interactions with the brand. As an Oatly representative discussed of its advertising campaign in the London Underground, this consumer interaction with the brand is unprecedented.

We might view the palatable disruption presented in claims for a post-milk generation as premised on a mode of what Goodman et al (2016) term ‘spectacular environmentalism’. This concept develops Guy Debord’s analysis of the rise of the ‘society of the spectacle’, in which ‘visual commodity fetishism’ supplants ‘real forms of human connection and sociality’ (Goodman et al 2016, p. 678). Goodman et al. apply this work to present modes of green-mediated consumption to help understand how consumers reflexively engage with advertising, especially on interactive social media. Mylks offer one such spectacular environmentalism. Here mylk becomes a green commodity fetish: an object alienated from the social and ecological relations of its production.

This fetishism is displayed in how companies engage with questions of sustainability and wellness. The environmental promises made of mylks often center on *outcomes* ascertained via life cycle assessments (LCAs). The rise of mylk is thus linked to the incursion of scientific expertise—both health claims from nutritional sciences and environmental claims from LCAs—into domestic spaces. Through these mechanisms, food companies posture as scientific experts through food choice. These calculations furthermore dissuade consideration of sustainability as a dynamic social-environmental *process* that involves multiple actors and locations. Seeing sustainability as an outcome rather than a process encourages technological fixes and standards (such as organic) to govern at a distance. These have been demonstrated to undermine attempts to improve environmental outcomes through food production-consumption (Guthman 2004; Mansfield 2004). Corporations, which excel at incremental technological changes (Goldstein 2018), have seized in mylks an opportunity to write themselves as heroes in food system change. In coordinating the green fetishizing of mylk, brands perform a spectacular form of environmental care.

This fetishism is similarly articulated in the health and wellness claims of mylk. Underlying these claims are at times specific statements about the nutritional qualities of mylk that are bolstered discursively by nutritional sciences and materially through supplementary injections of calcium, fats, and nutrients. At other times, health claims are tied to a relatively vague notion of wellness that is upheld more by what is absent in mylks; often dairy, soy, or additives. In selling wellness attached to convenience, consumers are puzzlingly encouraged to cure the negative psychological effects of a societal drive towards hyper-productivity by consuming an on-the-go product that enables them to continue to be ultra-productive. A carton of Califia Farms’ Ubermilk captures this with a thank-you note to itself: ‘thank you Ubermilk for being so extra. You go ABOVE and BEYOND so we can too.’ As with environmental claims, these health framings of mylks evoke a spectacle of care. This form of care through industry relies upon a neoliberal consumer-subject that desires to use food to cope with environmental catastrophe and a life out of balance.

These framings have served to remove mylks from social-ecological contexts. Mylks do not engender consumer connections with specific places, landscapes, farmers, environments, or animals. This represents a significant departure from the raft of AFNs that arose with an explicit mission of contesting placeless agri-industrial food by rebuilding trust through embedding food systems in places, as with local food movements (DuPuis and Goodman 2005) or connecting consumers to distant producers, as in fair trade (Goodman 2004). With mylks, the consumer relationship ends at the brand. This keeps politics firmly within the realm of consumption and power with corporations. Moreover, while AFNs such as organic entail standards and verification to regulate production practices (Guthman 2004), mylks are verified simply by the absence of animal products. As a result, prospects for governance of food production rely upon existing agricultural laws or the discretion of food companies.

In these ways, mylks sustain undemocratic production-consumption dynamics. Consumers are encouraged to disrupt their patterns by choosing foods marketed as better for their health and the environment. Yet, despite the premiums paid for mylks, these products often rely on commodity production systems that uphold the market logics embedded in late agrarian capitalism. While some mylk companies devote time to verifying the source of their plant ingredients, these products have added premiums. The bulk of mylk sales accrues to large companies that purchase ingredients on commodity markets. And, while commodity markets may be under increasing pressure to become more sustainable, environmental regulation through markets has inherent limits (Freidberg 2018). Almond milk, the continued leader among mylks, is a key example of these limits. More than 80% of global almond production occurs in drought-prone areas of California on mega-farms in monoculture systems. These systems have drained aquifers during droughts to irrigate almonds (Reisman 2019) and use copious herbicides (most notably glyphosate), which have contributed to the decimation of honeybee populations (McGivney 2020). These industrial almond production systems supply world-leading brand Almond Breeze and Silk Almondmilk, which together grossed just under $1 billion in US sales in 2019 (Shahbandeh 2019). Such agro-industrial production systems are effectively hidden with the claims of alternativeness and disruption discussed above.

## Nature’s perfect neoliberal food

This paper assessed how plant mylks have been de-politicized and naturalized as solutions to problems of climate change, animal welfare, and human health. Mylks write new chapters in what DuPuis (2002) has called the ‘perfect stories of milk,’ or the narratives of degradation and salvation that have been foundational in middle-class efforts of social reform since the nineteenth century. The ‘downfall’ story highlights the deleterious effects of industrial animal agricultural systems on the environment and human health. The ‘salvation’ narrative stars plant-based as a promise to cope with both environmental catastrophe and psychological distress of a hectic work life where there is little time to pause for meals. Mylks address this confluence of environment and health concerns by doubling down on the individual as the locus of change; effectively neoliberalizing governance of global environmental and public health issues.

With a historical study of milk in the US, DuPuis (2002) has demonstrated how middle-class social reformers, dairy farmers, politicians, and health experts worked together to frame milk as ‘nature’s perfect food’ in the twentieth century. This article depicts a similar politics of perfection and purification that serves to make mylks palatable in the twenty-first century. In contrast to the Fordist political economic structures undergirding dairy milk’s becoming a perfect food (DuPuis 2002), plant mylks are enmeshed in a neoliberal political economy. In offering a way to purchase imaginaries of wellness and climate change mitigation, mylks promote a neoliberal ethic of care. Like other forms of green consumerism, mylks identify the individual as the key actor and global markets as the platform for solving environmental and health problems. A politics of perfection depoliticized milk by attaching it to powerful social narratives of purity (DuPuis 2002). Similarly, by curating palatability and a food ethic based on absence, mylks depoliticize what might be a contested terrain of food system change. This serves to reinforce the political economy of agro-industry. Mylks thus appear to be a neoliberal articulation of food perfection. By strengthening corporate control over ‘alternatives’, mylks risk foreclosing on other potential pathways of food system change. As Guthman (2008, p. 442) suggests of AFNs in California, the mainstreaming of veganism through mylks reflects a ‘limited politics of the possible’.

Our aim in this article has been to open discussion to the limits of the current plant-based trend in hoped-for transitions to more just, ethical, and sustainable food futures. Our analysis traced how palatable disruption was achieved, identifying the importance of affective continuity in users experience of milk, the role played by cozy marketing to flexitarians, and the importance of spectacular modes of green commodity fetishism. As others put forth, mylks do have disruptive possibilities (Gambert and Linné 2019). Yet, a ‘post-milk’ future will not automatically address problems caused by the overproduction of industrial foods. Mylks excel in their ability to make food placeless. With further legitimacy gained from nutritionism and LCAs—and without animals getting in the way—mylk may be even more effective as a commodity than dairy milk. By merely grafting plant milks into existing production-consumption practices, agro-industrial problems are not so much fixed as they are diverted, obscured, or even forgotten. Mylks may afford at best an interruption to the challenges they claim to resolve. At worst, they could distract from the need for systemic changes by virtue of fitting so well within the contours of globalized industrial agri-food.

Increased consumption of plant mylk could in theory drive change in dairy systems through decreased demand for dairy milk. Yet such a trajectory is far from given. Dairy systems are highly heterogeneous (Clay et al. 2020). Water use, land use, and greenhouse gas emissions various enormously across farms and regions (Poore and Nemececk 2018). A post-milk imaginary does not necessarily exert influence over the type of dairy system. If past trajectories of intensification in the dairy sector are an indication, a likely response to decreased milk demand could be for the industrial dairy industry to further intensify production. Even though fluid milk consumption is decreasing in the US and Europe, it is increasing worldwide. One possible outcome is that mylk consumption will encourage industrial dairy systems that are environmentally harmful and of limited benefit to rural livelihoods. Continued consolidation into mega-farms has been driven in the past by price competition that privileges economies of scale. At the same time, dairy operations with a lower environmental footprint, higher animal welfare, and value to rural livelihoods and cultural landscapes will likely continue disappearing.

This interpretation of the politics of plant-based milk is meant as cautionary rather than dismissive. Plant-based milk and meat are flourishing. As these products to grow and diversify, it is crucial to consider how they might enable more democratic food futures. Flexitarianism presents a potentially open, inclusive, and democratic form of consumption that could drive food system change in just and sustainable ways. Its crux may be its mutability, which makes it readily co-optable. The corporate mylk regime that was the focus of this article does not exhaust the cultural, political, and economic forms that configure how milk alternatives can and do arise. Much less does it encapsulate the pathways by which we might transition to plant-rich diets. This is the crucial point. Despite this industrial incarnation, plant mylk can in fact be made at home with relative ease (at least compared to dairy milk, which requires a lactating mammal). There is no necessary reason why liquids derived from plants cannot give rise to environmentally beneficial, socially just, ethical, and nutritious ways of feeding people. Yet, assuring that they do requires attention to processes of production, distribution, and consumption.

## Footnotes

1 Since 2013, as a result of pressure from the dairy sector, EU regulations have stated that designations like milk, butter, cheese, cream and yogurt can only be used to market products derived from animal milk. Plant milk companies have responded with a set of neologisms including ‘mylk’ (Rebel Kitchen), ‘m*lk’ (Minor Figures) and ‘malk’ (Malk Organics). We use mylk in this paper as shorthand for the range of plant-based milk alternatives. The term also captures the industry’s vision of the ‘disruptive possibilities’ of these beverages (Gambert and Linné 2019, p. 65). We recognize that in using this term we may be subtly reinforcing its visibility.

2 Vertical integration in the milk sector has given retailers power to set milk prices at levels that are substantially lower than the cost of production for most smaller farms (Jay and Morad 2007; MacDonald et al. 2016).

3 Infant mortality was particularly high in urban areas in the US during this time, with diarrhoea often blamed on bacteria in milk, as well as other diseases such as strep throat and tuberculosis (Freidberg 2009).

4 These links continue to be the subject of vigorous dispute.

5 See also a recent interview with Califia Farms (almond milk company) Greg Stentenpohl, in which he discusses the importance of what he calls ‘flavor cues’: https://www.foodnavigator-usa.com/Article/2019/11/15/PepsiCo-drops-Quaker-Oat-Beverage-less-than-a-year-after-launch.

6 The trope of overcoming privileged ‘adversity’ is represented, for instance, in the You and Almonds Vs. marketing campaign by the California Almond Board, in which almonds provide the strength to fix a printer out of toner or locate a misplaced television remote control.

7 This is also true for soya, where European (over US or South America) provenance is coded as an environmental good due to perceived GM-free status.

8 By affective style we are referring to the emotional tenor of the advertisement that is produced through the planned juxtaposition of sound and imagery, alongside spoken and written content.

9 Chantel Mouffe (2013) and Jacques Rancière (2010), for example, argue that politics requires disagreement, claiming that dissensus is necessary for robust and accountable decision making.
